# 12 Weeks of Combined Endurance and Resistance Training Reduces Innate Markers of Inflammation in a Randomized Controlled Clinical Trial in Patients with Multiple Sclerosis

**DOI:** 10.1155/2016/6789276

**Published:** 2016-01-20

**Authors:** Nathalie Deckx, Inez Wens, Amber H. Nuyts, Niel Hens, Benedicte Y. De Winter, Gudrun Koppen, Herman Goossens, Pierre Van Damme, Zwi N. Berneman, Bert O. Eijnde, Nathalie Cools

**Affiliations:** ^1^Laboratory of Experimental Hematology, Vaccine & Infectious Disease Institute (VAXINFECTIO), Faculty of Medicine and Health Sciences, University of Antwerp, 2610 Wilrijk, Belgium; ^2^REVAL Rehabilitation Research Centre, BIOMED Biomedical Research Institute, Faculty of Medicine and Life Sciences, Hasselt University, 3590 Diepenbeek, Belgium; ^3^Interuniversity Institute for Biostatistics and Statistical Bioinformatics (I-BIOSTAT), Hasselt University, 3590 Diepenbeek, Belgium; ^4^Centre for Health Economic Research and Modelling Infectious Diseases (CHERMID), Vaccine & Infectious Disease Institute (VAXINFECTIO), Faculty of Medicine and Health Sciences, University of Antwerp, 2610 Wilrijk, Belgium; ^5^Laboratory of Experimental Medicine and Pediatrics, Division of Gastroenterology, Faculty of Medicine and Health Sciences, University of Antwerp, 2610 Wilrijk, Belgium; ^6^Flemish Institute for Technological Research (VITO), Environmental Risk and Health Unit, 2400 Mol, Belgium; ^7^Laboratory of Medical Microbiology, Vaccine & Infectious Diseases Institute (VAXINFECTIO), Faculty of Medicine and Health Sciences, University of Antwerp, 2610 Wilrijk, Belgium; ^8^Centre for the Evaluation of Vaccination, Vaccine & Infectious Disease Institute (VAXINFECTIO), Faculty of Medicine and Health Sciences, University of Antwerp, 2610 Wilrijk, Belgium

## Abstract

Previously, we reported that patients with multiple sclerosis (MS) demonstrate improved muscle strength, exercise tolerance, and lean tissue mass following a combined endurance and resistance exercise program. However, the effect of exercise on the underlying disease pathogenesis remains elusive. Since recent evidence supports a crucial role of dendritic cells (DC) in the pathogenesis of MS, we investigated the effect of a 12-week combined exercise program in MS patients on the number and function of DC. We demonstrate an increased number of plasmacytoid DC (pDC) following the exercise program. These pDC display an activated phenotype, as evidenced by increased numbers of circulating CD62L^+^ and CD80^+^ pDC. Interestingly, the number of CD80^+^ pDC positively correlates with the presence of IL-10-producing regulatory type 1 cells (Tr1), an important cell type for maintaining peripheral tolerance to self-antigens. In addition, decreased production of the inflammatory mediators, TNF-*α* and MMP-9, upon Toll-like receptor (TLR) stimulation was found at the end of the exercise program. Overall, our findings suggest that the 12-week exercise program reduces the secretion of inflammatory mediators upon TLR stimulation and promotes the immunoregulatory function of circulating pDC, suggestive for a favorable impact of exercise on the underlying immunopathogenesis of MS.

## 1. Introduction

Multiple sclerosis (MS) is a chronic demyelinating, inflammatory disease of the central nervous system (CNS), predominantly affecting young adults in their most productive years. It is a heterogeneous disease with regard to both its clinical course and symptoms. The most prevalent symptoms are sensory changes, including fatigue, balance disorders, spasticity, motor weakness, and impaired muscular performance [1]. Besides incremental disability, these symptoms also result in a reduced quality of life. Nevertheless, a considerable part of MS symptoms may also result from a sedentary lifestyle [2]. Over the years, different studies have reported improvement in muscle strength [3], cardiovascular health [4], mental functioning [2], and quality of life [5] following a physical exercise intervention in MS patients. Recently, we demonstrated improvement in muscle strength, exercise tolerance, and lean tissue mass in MS patients following a combined endurance and resistance exercise program [6]. Although it is likely that only the effects of a sedentary lifestyle can be reversed by physical exercise [7], it has been suggested that physical exercise might have the potential to have an impact on the disease pathogenesis of MS as well [8]. To date, however, little is known about the potential effects of physical exercise on the underlying disease mechanisms.

Although the exact cause of MS is still unknown, it is now generally accepted that MS is a complex multifactorial disease involving genetic and environmental factors affecting the autoreactive immune response [9]. Based on previous efforts focusing on the role of the adaptive immune system in the pathogenesis of MS, it is currently well established that autoreactive T helper type 1 (Th1) and Th17 cells mediate the inflammatory processes in the CNS [10]. Recent evidence also suggests involvement of innate immunity, including dendritic cells (DC), in the initiation and maintenance as well as progression of MS [11].

DC are professional antigen-presenting cells with the unique capacity to polarize the differentiation of T cells, thereby regulating the balance between inflammation and tolerance [12]. To perform this function, DC are capable of capturing antigens, processing them, and presenting them on the cell surface complexed to major histocompatibility (MHC) molecules, for example, human leukocyte antigen- (HLA-) DR. Along with that, context-dependent expression of costimulatory molecules, such as CD80 and CD86, and secretion of cytokines occurs. In human blood, two major subsets of DC have been characterized, that is, myeloid or conventional DC (cDC) and plasmacytoid DC (pDC) [13]. Both DC subsets display a different expression profile of cytokine receptors and cytokines, of migratory markers and migration potential, and of Toll-like receptors (TLR). In brief, cDC strongly express TLR2 and TLR4, among other TLR, which are stimulated by bacterial triacylated lipopeptides, Gram-negative bacterial lipopolysaccharide (LPS), fungal mannans, parasitic phospholipids, viral envelope proteins, and host-derived heat shock proteins. Upon activation, cDC secrete proinflammatory cytokines such as interleukin- (IL-) 1*β*, IL-6, IL-12p70, and tumour necrosis factor- (TNF-) *α* [13]. In addition, cDC also secrete the matrix metalloproteinase- (MMP-) 9, a protease that is involved in the breakdown of extracellular matrix in normal physiological processes, including cell migration [14]. On the other hand, pDC express TLR7 and TLR9, which are stimulated by single-stranded RNA and unmethylated CpG oligodeoxynucleotides. Upon activation, pDC secrete significant amounts of type I interferon (IFN) [15]. Depending on their costimulatory molecule expression and cytokine secretion, both cDC and pDC are able to induce effector T cells as well as regulatory T cells (Treg) [12]. While in a steady state, both cDC and pDC are poor T cell stimulators, given their low expression levels of MHC and costimulatory molecules, activated cDC and pDC differ strongly in their T cell-stimulatory capacity. Following TLR ligation, activated cDC efficiently stimulate effector T cells as they upregulate the expression of costimulatory molecules and cytokines. On the other hand, TLR-mediated recognition of viral products and RNA/DNA/immunocomplexes by pDC results in the production of large amounts of type I IFN [15]. For this, pDC effectively participate in antiviral immunity. Nevertheless, besides this protective role, pDC may also play a role in aberrant immune responses. For instance, it has been suggested that pDC promote the progression of systemic lupus erythematosus following sensing of self-DNA and subsequent production of type I IFN [16]. In contrast, pDC have been implicated in maintenance of tolerance. It has been reported that pDC contribute to peripheral T cell tolerance in transplantation [17], tumour escape [18], oral tolerance [19], and mucosal tolerance [20]. Indeed, pDC display tolerogenic functions with the ability to indirectly suppress T cell proliferation through the induction of Treg [21].

Because of their unique capacity to shape immune responses [12], DC are thought to play a pivotal role in the immunopathogenesis of several autoimmune disorders, including MS. Previously, we reported an aberrant frequency and expression of costimulatory markers on DC in the peripheral blood of MS patients. Circulating DC also display divergent expression of migration markers in MS patients as compared to DC from healthy controls. In addition, we demonstrated increased secretion of IL-12p70 by LPS- and IFN-*γ*-stimulated DC from relapsing-remitting (RR) MS patients as compared to healthy controls [22]. Furthermore, upon stimulation with TLR9 ligands, IFN-*α* secretion by pDC from RRMS patients is reduced as compared to the level secreted by pDC from healthy controls [23].

Previously, others demonstrated that physical exercise alters the number and function of leukocytes, including circulating cells of the innate immune system, such as neutrophils, monocytes, and natural killer cells [24]. However, the effect of physical exercise on DC is rather unexplored. Increased DC numbers were found following exercise training in an animal model, whereas their phenotype, that is, the expression of the costimulatory molecules CD80 and CD86 by DC, was not affected [25]. Interestingly, after 5 weeks of physical exercise, bone marrow-derived rat DC displayed a more pronounced immune-stimulatory function [25]. Also in humans, increased numbers of cDC, but not pDC, were found following exercise [26, 27].

Altogether, it has been demonstrated that physical exercise is well tolerated and induces relevant improvements in both physical and mental functioning of MS patients, although its effect on underlying mechanisms remains to be elucidated. Since recent evidence suggests a crucial role for innate immunity in the initiation as well as progression of MS, we aim to investigate the effect of a 12-week combined endurance and resistance exercise program on cellular and molecular mediators of innate immunity in MS patients.

## 2. Material and methods

### 2.1. Study Population

A total of 67 MS patients, diagnosed according to the revised McDonald criteria [28] and aged >18 years, were assessed for eligibility by the REVAL Rehabilitation Research Centre. Subjects were excluded if they had an expanded disability status scale (EDSS) score >6, that is, not able to walk 100 m without walking aid, physician-diagnosed diabetes mellitus type II, other autoimmune diseases (diabetes mellitus type I and/or rheumatoid arthritis), or other chronic diseases (cardiovascular, pulmonary, and/or renal), were pregnant, participated in another study, had contraindications to perform physical exercise, received corticosteroid treatment 3 months prior to the study, or had an acute MS exacerbation 6 months prior to the study. All subjects gave informed consent in accordance with the declaration of Helsinki and the protocol was approved by the local Ethics Committee of Virga Jesse Hospital, Hasselt, and Hasselt University and registered at https://www.clinicaltrials.gov/ (NCT01718392 and NCT01845896).

### 2.2. Study Design

**Figure 1:**
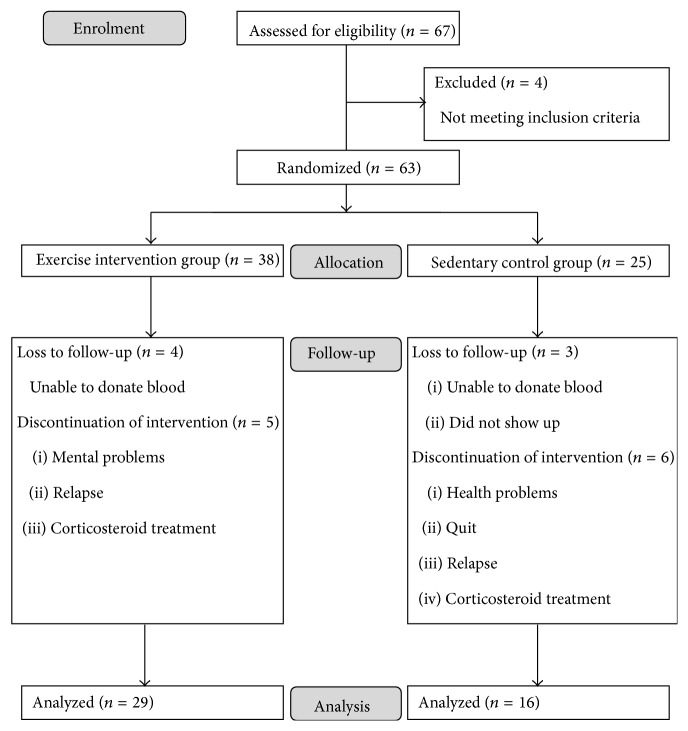
Flow diagram of patient enrolment.

MS patients were randomized by sealed envelope following a 3 : 2 ratio to an exercise intervention group (EX: *n* = 38) or a sedentary control group (SED: *n* = 25) by the REVAL Rehabilitation Research Centre. Respectively, only 29 and 16 patients were included in the repeated measures analysis, due to loss to follow-up or discontinuation of intervention (Figure 1). The ratio of patients who discontinued the intervention and the ratio of patients who were lost to follow-up were not significantly different between the exercise and the sedentary group (see Supplementary Appendix in Supplementary Material available online at http://dx.doi.org/10.1155/2016/6789276).

Patient characteristics and medication use are depicted in Supplementary Table 1. Due to the nature of the trial, patients and caregivers could not be blinded to group allocation, whereas those assessing the outcomes were blinded to group allocation. Patients in the exercise group participated in a supervised 12-week combined endurance and resistance training program in the REVAL Rehabilitation Research Centre at a frequency of 5 sessions per 2 weeks. Each session started with a cardiovascular part, consisting of cycling and treadmill walking or running (Technogym, LJ Capelle aan den Ijssel, The Netherlands). Session duration and intensity increased as the program proceeded, starting from 1 × 6 min/session to 3 × 10 min/session, interspersed by 2-3 minutes of rest. The duration was determined beforehand, whereas the intensity was determined individually depending on heart rate and disability level. For example, participants needed to be able to walk/pedal uninterrupted and independently for the prescribed minutes, and intensity decreased when they had difficulties keeping up with the pace or felt not confident to succeed in the program. The second part consisted of resistance training (leg press, leg curl, leg extension, vertical traction, arm curl, and chest press, Technogym). Resistance training of the lower limb was performed unilaterally, due to bilateral strength differences between the legs of MS patients. To improve muscle fitness, sets of repetitions gradually increased during intervention, from 1 × 10 repetitions to 4 × 15 repetitions, with maximal attainable load, interspersed by 1-2 minutes of rest. The number of repetitions was determined beforehand, whereas the intensity was determined individually, based on individual disability levels, which are inconstant in MS. When the number of repetitions increased, the maximal attainable load decreased. After two or three sessions, working with the same number of repetitions, participants were encouraged to, if possible, increase their weights again. Continuous encouragement and supervision by the instructors during the training period led to a systematic increase of the training load over the 12-week training period. The sessions were finished by stretching. Patients in the sedentary group did not participate in any training program and were asked to continue standard care. Blood (20 mL) was sampled from patients in both groups in fasting state at 8 a.m. following abstinence from exercise for 48 hours. Samples were collected before the combined exercise program (PRE) and within one week after the end of the 12-week training program (POST) in both heparin and serum-separating tubes (BD Biosciences, Erembodegem, Belgium).

### 2.3. Isolation and Stimulation of Leukocytes

Leukocyte cell counts were measured using an automated cell counter (ABX Micros 60, Horiba, Deurne, Belgium). Next, leukocytes were isolated for* ex vivo* flow cytometric analysis of DC and Treg subsets using density gradient purification (Ficoll Paque PLUS, GE Healthcare, Chalfont St Giles, UK). In addition, for evaluation of Treg frequencies using intracellular flow cytometry, leukocytes were treated overnight with brefeldin A (10 *µ*g/mL) (Life Technologies, Merelbeke, Belgium). Simultaneously, 1 mL of peripheral blood was (i) stimulated overnight with 2 *μ*g/mL LPS, a TLR4 ligand (Invivogen, Toulouse, France), and 50 *µ*g/mL IFN-*γ* (Immunotools, Friesoythe, Germany) or (ii) stimulated overnight with 10 *μ*g/mL imiquimod (IQ), a synthetic TLR7 ligand (Invivogen) or (iii) left untreated as a control. For evaluation of TLR-mediated cytokine production, plasma was collected and stored at −20°C for further use in cytokine secretion assays. For evaluation of DC activation state, leukocytes were enriched after red blood cell lysis (0.155 M NH_4_Cl, 0.01 M KHCO_3_, and 0.1 mM Na_2_-EDTA).

### 2.4. Flow Cytometry

Immunophenotyping of DC was done by direct immunofluorescence staining using the following fluorochrome-labeled mouse anti-human monoclonal antibodies: anti-blood dendritic cell antigen- (BDCA-) 1 phycoerythrin (PE) (Miltenyi Biotec, Leiden, The Netherlands), anti-BDCA-2 allophycocyanin (APC) (Miltenyi Biotec), anti-lineage I (Lin I; anti-CD3, anti-CD14, anti-CD16, anti-CD19, anti-CD20, and anti-CD56) fluorescein isothiocyanate (FITC) (BD Biosciences), anti-CD62L PE-cyanine 7 (PE-Cy7) (eBioscience, Vienna, Austria), anti-CD80 APC-H7 (BD Biosciences), anti-CD86 V450 (BD Biosciences), anti-CCR5 PE-Cy7 (BD Biosciences), anti-CCR7 V450 (BD Biosciences), and anti-human leukocyte antigen- (HLA-) DR APC-H7 (BD Biosciences) antibodies. Moreover, dead cells were excluded by addition of violet live/dead stain (Life Technologies) to the antibody mixture. cDC and pDC were identified as Lin I^−^ BDCA-1^+^ and Lin I^−^ BDCA-2^+^, respectively. The number of DC displaying an activated or a migratory phenotype was assessed by enumerating cDC and pDC expressing costimulatory molecules (CD80, CD86) and migration markers (CD62L, CCR5, and CCR7), respectively.

Treg subsets were characterized using the following fluorochrome-labeled mouse anti-human monoclonal antibodies: anti-CD3 peridinin chlorophyll protein Cy5.5 (PerCP-Cy5.5) (BD Biosciences), anti-CD4 APC-H7 (BD Biosciences), anti-CD8 pacific blue (PB) (Life Technologies), anti-CD25 PE-Cy7 (BD Biosciences), anti-IL-10 APC (BD Biosciences), anti-transforming growth factor- (TGF-) *β* PE (IQ Products, Groningen, The Netherlands), and anti-forkhead box P3 (FoxP3) Alexa Fluor 488 (BD Biosciences). Moreover, dead cells were excluded by addition of violet live/dead stain (Life Technologies) to the antibody mixture. Naturally occurring Treg were identified as CD25^hi^FoxP3^+^, while induced Treg, that is, regulatory type 1 cells (Tr1) and T helper 3 (Th3) cells, were characterized based on the production of the anti-inflammatory cytokines IL-10 and TGF-*β*, respectively.

Fluorescence minus one in combination with nonreactive isotype-matched antibodies was used as control. For analytical flow cytometry, at least 10^5^ events were measured using a three-laser instrument configured to measure up to 15 parameters simultaneously, that is, a Cyflow ML flow cytometer (Partec, Münster, Germany). All results were analyzed using FlowJo software (Tree Star, Inc., Ashland, OR, USA).

### 2.5. Soluble Analyte Secretion Assays

Serum was isolated using serum-separating tubes (BD Biosciences). Quantitative detection of cortisol (Alpco, Salem, NH, USA) and dehydroepiandrosterone (DHEA; Alpco), was determined by means of ELISA according to manufacturer's instructions. Levels of the primary human anti-inflammatory glucocorticoid, cortisol, are often insufficient to evaluate the function of the hypothalamus-pituitary-adrenal (HPA) axis. Therefore, we also quantified the serum concentration of DHEA, a precursor of the sex hormones testosterone and estrogen and a glucocorticoid antagonist, in order to calculate the molar ratio of cortisol to DHEA [29, 30]. In addition, serum levels of IL-6, IL-10, IL-12p70, TNF-*α*, MMP-9, and the inflammasome effector molecule caspase-1 (R&D, Minneapolis, MN, USA) were quantified using ELISA according to manufacturer's instructions. All kits were purchased from Meso Scale Discovery (MSD, Rockville, MD, USA), unless stated otherwise. For quantitative detection of the cytokines secreted following stimulation of peripheral blood, collected plasma samples were analyzed using the following commercially available ELISA kits: IL-1*β*, IL-6, IL-12p70, TNF-*α*, IFN-*α* (PBL InterferonSource, Piscataway, NJ, USA), caspase-1 (R&D), and MMP-9, according to manufacturer's instructions. All kits were purchased from eBioscience, unless stated otherwise.

### 2.6. Statistical Analysis

All data were analyzed using SAS 9.3 software (SAS Institute Inc., Cary, USA). Linear mixed models were used to analyze repeated measures data [31]. A model was built stepwise per outcome variable, starting from a univariate model with “time” as the only fixed effect. Step by step new models were constructed by adding other fixed effect variables including group, MS type, gender, age, body mass index (BMI), EDSS, and MS-specific medication. The variable “group” included trained (EX) and sedentary MS patients (SED). The variable “MS type” included relapsing-remitting (RR) and chronic-progressive (CP) MS patients. The variable “MS-specific medication” included untreated 1st-line treatment and 2nd-line treatment. A *P* value < 0.10 (*F* test) was used as the threshold for retaining a fixed effect during model building to decrease the chance of missing a significant effect in the final model. Subsequently, possible interaction effects between the retained fixed effects were assessed and also retained if *P* < 0.10. For interpretation of the fixed effects in the final model, the threshold for statistical significance was set at *P* < 0.05. Since we aim to focus on temporal effects, we only report main and interaction effects of time. Furthermore, since our main interest is the effect of time, we did not include baseline measurements of patients who did not complete the study, that is, patients who were lost to follow-up or patients who discontinued the intervention. No bias was expected for excluding baseline measurements from patients who did not complete the study (Supplementary Appendix). Nevertheless, to determine safety of the intervention, patients who relapsed during the study were noted and the annualized relapse rate was calculated for both groups. Models consisting of more than 12 parameters were not interpreted because of the risk of overfitting. With significant interaction effects of time, post hoc analyses were performed using the Bonferroni correction to correct for multiple testing. Moreover, based on our a priori hypotheses that an effect of time, that is, the 12-week exercise intervention, could be observed in the trained group of patients and not in the sedentary group of patients [24], post hoc analyses for evaluating the effect of time in both the trained and the sedentary group were always performed, thereby reducing the chance of false negative results. Moreover, we used the conservative Bonferroni correction to account for multiple testing, thereby reducing the chance of detecting false positive results (Supplementary Appendix). Diagnostics were based on the studentized residuals and response variables were log-transformed when necessary. Finally, relevant correlations were assessed using Pearson's correlation test. Graphs were generated in SAS 9.3 software and GraphPad version 5 software (Prism, La Jolla, CA, USA). All data are presented as mean ± standard error of the mean (SEM).

## 3. Results

### 3.1. No Baseline Differences between Trained and Sedentary MS Patients

MS patients were randomized following a 3 : 2 ratio to an exercise intervention group (EX: *n* = 38) or a sedentary control group (SED: *n* = 25). Patients in the exercise group reached a mean adherence percentage of 90%, ranging from 80 to 100%, at the end of the 12-week training program. At the end of the study, all data points from 29 and 16 patients in the exercise and the sedentary group, respectively, were collected for analysis (Figure 1), changing the ratio to 2 : 1. No differences in the distribution of gender, age, BMI, clinical course of MS, EDSS, and use of MS-specific medication could be detected for the analyzed patients between both study groups (Table 1). Similarly, no differences at baseline could be detected for all experimental outcomes tested between the exercise group and the sedentary control group except for the number of CCR5^+^ cDC (*P* = 0.033), which was higher in the exercise group (*vide infra*).

### 3.2. Serum Signaling Molecule Concentrations Are Not Altered following Long-Term Physical Exercise in MS

Although no difference in the cortisol concentration between the patient groups in the course of the 12-week time period could be detected, cortisol levels significantly increased in the trained group after the 12-week combined exercise program (*P* = 0.047, Table 2), while no change was observed in the sedentary control group. However, the physiological relevance of this relatively small increase (fold increase = 1.07) is debatable. No significant differences of the concentration of DHEA and the molar concentration of cortisol to DHEA could be detected following the 12-week time period between the exercise group and the sedentary control group. In our hands, no significant differences could be detected between the exercise group and the sedentary control group in the course of the 12-week time period with regard to the serum concentration of IL-6, IL-10, IL-12p70, TNF-*α*, caspase-1, and MMP-9 (Table 2).

### 3.3. Circulating pDC Numbers Increase following Long-Term Physical Exercise in MS

**Figure 2:**
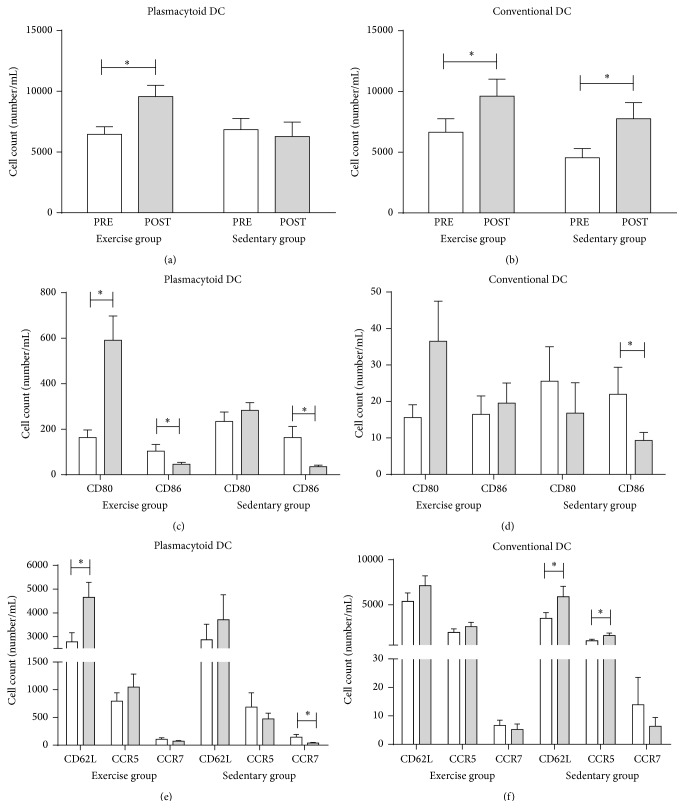
Increased numbers of activated pDC are found following a 12-week combined exercise program in MS. Enumeration of the absolute number of circulating pDC (a) and cDC (b) was determined by means of a double platform method using an automatic cell counter and flow cytometry before start of the study (open bars) and after 12 weeks (filled bars). The activation state (c, d) and the migratory profile (e, f) of DC were assessed by enumerating pDC and cDC expressing CD80, CD86, CD62L, CCR5, and CCR7. Results are shown as mean absolute number ± SEM. ^*∗*^ *P* < 0.05. cDC, conventional dendritic cells; pDC, plasmacytoid dendritic cells; EX, exercise group; SED, sedentary control group; SEM, standard error of the mean.

We were not able to detect an effect of the physical exercise program on the absolute number of total leukocytes nor on the absolute number of lymphocytes, monocytes, and granulocytes (data not shown). Next, we investigated the number of circulating DC subsets, that is, cDC and pDC, in MS patients participating in the combined exercise program (EX, *n* = 29) as compared to sedentary control patients (SED, *n* = 16). Our findings demonstrate that, in the course of the 12-week time period, a difference between trained and sedentary patients could be demonstrated regarding the absolute number of pDC (*P* = 0.017). Indeed, increased cell counts of pDC were detected in the exercise group (*P* = 0.010; Figure 2(a)) after 12 weeks, while no changes were observed in the sedentary control group. Also the proportion of circulating pDC within total white blood cells was increased in MS patients who participated in the exercise program (*P* = 0.050; data not shown). No effect of the 12-week intervention on the absolute number of cDC could be detected. Both patient groups displayed an increase in the absolute number of cDC after 12 weeks (EX: *P* = 0.026, SED: *P* = 0.002; Figure 2(b)).

### 3.4. Increased Numbers of pDC with an Activated Phenotype Are Found following the Physical Exercise Program in MS

The absolute number of cDC and pDC positive for the costimulatory molecules CD80 and CD86 was analyzed by flow cytometry. In the course of the 12-week time period, a difference was observed between the patient groups for the absolute number of CD80^+^ pDC (*P* = 0.015) and of CD86^+^ pDC (*P* = 0.016). Indeed, significantly increased numbers of CD80^+^ pDC were observed in MS patients following the 12-week combined exercise program (*P* < 0.001; Figure 2(c)), while no differences in the number of CD80^+^ pDC could be found in the sedentary control group. Similarly, also the proportion of circulating CD80^+^ pDC within total white blood cells was increased in MS patients who participated in the exercise program (*P* = 0.030; data not shown). Both intervention groups display, however, a decrease in CD86^+^ pDC numbers after 12 weeks (EX: *P* = 0.046, SED: *P* = 0.001; Figure 2(c)), indicative of an effect that cannot be attributed to the physical exercise program. In addition, no differences in the course of the 12-week time period could be detected between the patient groups for the absolute number of CD80^+^ and CD86^+^ cDC, although CD86^+^ cDC significantly decreased after 12 weeks in the sedentary control group (*P* = 0.022; Figure 2(d)).

Simultaneously, the absolute number of cDC and pDC positive for the migration markers, CD62L, CCR5, and CCR7, was measured. No differences regarding the absolute number of CD62L^+^, CCR5^+^, and CCR7^+^ pDC were detected between the patient groups in the course of the 12-week time period. However, we did show a significantly increased number of CD62L^+^ pDC in MS patients following the exercise program (*P* = 0.002; Figure 2(e)), while no changes were observed in the sedentary control group. In addition, a modest but significant decrease in the number of CCR7^+^ pDC could be detected after 12 weeks in the sedentary control group (*P* = 0.009; Figure 2(e)). No differences regarding the absolute number of CD62L^+^, CCR5^+^, and CCR7^+^ cDC could be detected between the groups in the course of the 12-week time period, although modest but significant changes in the number of CD62L^+^ and CCR5^+^ cDC could be observed in the sedentary control group (CD62L: *P* = 0.041, CCR5: *P* = 0.025; Figure 2(f)).

Taken together, our observations show that increased numbers of CD80^+^ and CD62L^+^ pDC, indicative of an activated phenotype, are found following the exercise program.

### 3.5. TLR Responsiveness following Long-Term Physical Exercise in MS Is Altered

In order to assess the responsiveness of circulating DC to danger signals, blood samples were stimulated with a TLR4 ligand, LPS in combination with IFN-*γ*, or a TLR7 ligand, IQ. Both ligands are known to activate cDC and pDC, respectively [32, 33]. Following 12 weeks of exercise, the fold change of CCR5^+^ cDC upon LPS and IFN-*γ* stimulation was decreased in the exercise group (*P* = 0.002; Table 3) and significantly differed from the sedentary group in the course of the 12-week time period (*P* = 0.038), where no changes were observed. No differences regarding TNF-*α* and MMP-9 secretion upon LPS and IFN-*γ* stimulation could be detected between the patient groups in the course of the 12-week time period. Nevertheless, TNF-*α* and MMP-9 secretion was significantly decreased in trained MS patients (TNF-*α*: *P* = 0.028; MMP-9: *P* = 0.040), while no changes were detected in the sedentary control group (Table 4). In addition, no significant differences could be detected for the fold change of CD86^+^ and HLA-DR^+^ cDC, neither for the secreted amount of IL-1*β*, IL-6, IL-12p70, IFN-*α*, and caspase-1 upon LPS and IFN-*γ* stimulation between the trained and sedentary MS patients after 12 weeks (Tables 3 and 4).

In the course of the 12-week time period, the fold change of HLA-DR^+^ pDC upon IQ stimulation differed significantly between the patient groups (*P* = 0.023). A significant increase in the fold change of HLA-DR^+^ pDC upon IQ stimulation was observed in MS patients after 12 weeks of exercise (*P* = 0.008), but not in the sedentary control group (Table 3). Furthermore, IL-1*β* secretion upon IQ stimulation differed between the patient groups in the course of the 12-week time period (*P* = 0.039, Table 4). Nevertheless, after 12 weeks, IL-1*β* secretion was only significantly decreased in the sedentary control group (*P* = 0.029), indicative of an effect that cannot be attributed to the physical exercise program. No significant differences could be detected for the fold change of CCR5^+^ and CD86^+^ pDC nor for the secreted amount of IL-6, IL-12p70, TNF-*α*, IFN-*α*, caspase-1, and MMP-9 upon IQ stimulation between trained and sedentary MS patients after 12 weeks (Tables 3 and 4).

### 3.6. Circulating Treg Subsets Are Not Affected by Long-Term Physical Exercise in MS

Using flow cytometry, we next characterized the effect of physical exercise on Treg subsets, that is, CD25^hi^FoxP3^+^, Tr1, and Th3 (Table 5).

**Figure 3:**
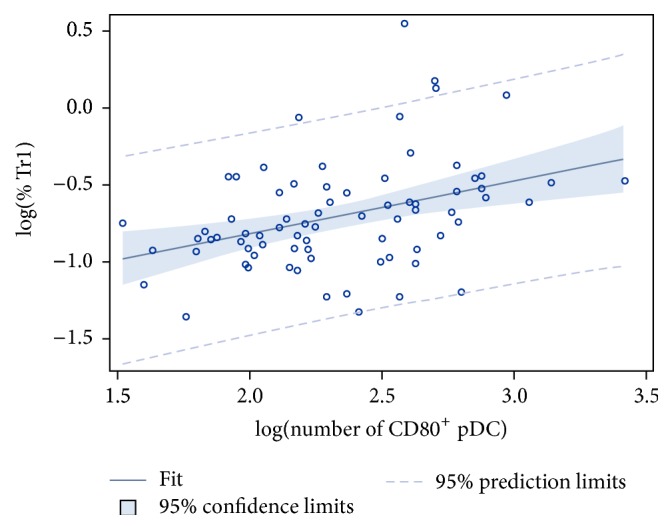
Positive correlation at log-log scale between the proportion of Tr1 and the number of CD80^+^ pDC. A positive correlation at log-log scale was found between the proportion of Tr1 cells and the number of CD80^+^ pDC (*P* = 0.001; *ρ* = 0.383). Both variables were log-transformed to obtain a normal distribution. pDC, plasmacytoid dendritic cells; Tr1, type 1 regulatory T cell; log, logarithmic transformation; *ρ*, Pearson's correlation coefficient.

In our hands, no effect of the 12-week combined exercise program on the proportion of Treg populations could be detected. Similarly, no effect was observed in the sedentary control group. Interestingly, a positive correlation was found at log-log scale between the proportion of IL-10-producing Tr1 cells and the number of CD80^+^ pDC (*P* = 0.001, *ρ* = 0.383; Figure 3).

## 4. Discussion

Based on previously demonstrated improvements in muscle strength, cardiovascular health, mental functioning, and quality of life in MS patients participating in an exercise program, we examined the modulation of immune variables that are known to impact disease activity in MS, namely, cellular and molecular mediators of innate immunity, following a 12-week combined exercise program in this study. This hypothesis is supported by studies investigating the effect of physical exercise in the experimental autoimmune encephalomyelitis (EAE) animal model for MS [8].

We were not able to detect differences following 12 weeks of exercise for the IL-6 and IL-12p70 serum concentration, similar to findings made by others [34–36]. Likewise, no differences in the serum concentration of IL-10, TNF-*α*, caspase-1, and MMP-9 were found following the exercise program. After an exercise program in MS patients, White et al. found decreased IL-10 serum levels [35], while TNF-*α* serum levels increased [36], decreased [35], or remained unchanged [34]. MMP-9 serum levels increased [37] or decreased [38] following an exercise program in healthy men and men with metabolic syndrome, respectively. The different outcomes of the various studies could be attributed to the variability in training protocols and study populations or to the different techniques used to quantify the secreted levels of inflammatory mediators.

Interestingly, we demonstrated decreased TNF-*α* and MMP-9 secretion following whole blood stimulation with LPS and IFN-*γ* in trained MS patients, which may be of potential interest in disease-mediated pathways. We hypothesize that the observed reduced secretion of TNF-*α* might contribute to a diminished stimulation of myelin-specific Th1 and Th17 effector cells, ultimately resulting in attenuated disease activity. In support of this, reduced frequencies of LPS- and IFN-*γ*-stimulated cytokine-producing monocytes and cDC were found in elite swimmers after long-term intensive training [39]. Furthermore, reduced LPS-induced cytokine production by monocyte-derived DC resulted in diminished stimulation of Th1 and Th17 effector cells [40]. In addition, the observed reduced secretion of MMP-9 might positively affect blood-brain barrier integrity. Indeed, the primary role of MMP-9 is degradation of the extracellular matrix, for example, during blood-brain barrier transmigration of leukocytes to the CNS, which is seen in MS [41]. Together with the significantly more pronounced reduction in CCR5^+^ cDC upon LPS and IFN-*γ* stimulation observed in MS patients who participated in the exercise program, this may suggest that exercise affects the migratory potential of cDC, although functional studies are needed to confirm this. In contrast to our findings, a reduction in LPS-induced secretion of IL-1*β*, IL-6, and IL-12 was observed after long-term physical exercise by others [39]. Nevertheless, further experiments are mandatory to identify the responsible cell types secreting the inflammatory mediators as well as to determine whether long-term exercise affects the surface expression of TLR resulting in the observed reduced secretion of inflammatory mediators. Indeed, whereas higher expression levels of TLR2 and TLR4 were found on mononuclear cells in MS patients as compared to healthy controls [42], a reduction in the expression levels of TLR could potentially mediate the favourable effect of physical exercise in MS patients.

No effect of exercise on the absolute number of cDC could be detected, as indicated by an increase in cDC numbers after the 12-week time period in both study groups. It has been demonstrated that changes in frequency of circulating myeloid cells correlate with certain chronic diseases [43]. Previously, we showed decreased numbers of circulating pDC in MS patients as compared to healthy controls [22]. Interestingly, in this study, increased cell counts of pDC, more specifically CD80^+^ pDC and CD62L^+^ pDC, were found after 12 weeks of exercise, indicative of an activated phenotype. However, others have reported conflicting results. Indeed, Chiang et al. found that healthy practitioners of Tai-Chi Chuan, an aerobic exercise of moderate intensity, showed increased numbers of circulating cDC, but not pDC, as compared to sedentary individuals [26]. Also in obese individuals an increased proportion of cDC, but a decreased proportion of pDC, was observed after 10 weeks of intensified exercise training [27]. Noteworthy, in the same study no differences regarding cDC and pDC numbers were observed in lean individuals. However, a sedentary control group was not included [27]. Others recently demonstrated that natalizumab treatment of MS also increases the number of circulating pDC [44]. However, in our hands no effect of natalizumab treatment on the reported findings could be observed (data not shown). Furthermore, we observed a significant increase in the fold change of HLA-DR^+^ pDC upon IQ stimulation after completion of the exercise program. This may suggest an increase in the number of pDC contributing to antigen presentation upon TLR stimulation in MS patients who participated in the exercise program.

Based on our observations demonstrating increased numbers of activated pDC following the 12-week combined exercise program as well as on the role of activated pDC in inducing IL-10-producing Treg (Tr1) [21], we hypothesized increased numbers of Tr1 in MS patients who completed the exercise program. Whereas in our hands no effect of long-term physical exercise on the proportion of Treg populations could be observed, others previously reported that individuals who participated in a long-term physical exercise program show higher proportions of circulating Treg [45]. Importantly, we demonstrate a positive correlation between the proportion of Tr1 and the number of circulating CD80^+^ pDC, underscoring the hypothesis that increasing numbers of activated pDC may drive the induction of Treg following long-term physical exercise in MS. However, the role of pDC in immunity and tolerance appears complex and activated pDC also participate in innate immunity with a specialized production of type I IFN. In doing so, pDC can stimulate the proliferation of naive T cells [46], albeit poorly relative to cDC. Nevertheless, no effect on the secretion of IFN-*α* by pDC upon TLR7 stimulation was observed following physical exercise in this study. Hence, it is less likely that increased activated pDC numbers induce effector T cell stimulation following long-term physical exercise, further supporting promotion of the immunoregulatory function of pDC. Future experiments are needed to understand the role of pDC in managing both innate and adaptive immune responses, for example, expression of inducible tolerogenic enzyme indoleamine 2,3-dioxygenase (IDO), inducible costimulator ligand (ICOS-L), and/or programmed death 1 ligand (PD-L1), which mediate Treg development, and how this is affected by long-term exercise. Additional prognostic studies addressing associations between immunologic effects of long-term exercise and clinical changes in MS patients are needed.

In this respect, it has been proposed that exercise stress influences the immune system via the activation of the HPA axis and the sympathetic nervous system, two main neuroendocrine pathways [47]. It is known that their respective signaling molecules, cortisol, DHEA, and catecholamines modulate the number, functioning, trafficking, and activity of immune cells. Hence, evaluating the hormonal release could offer insight into the mechanisms that underlie exercise-mediated immune modulation. Unfortunately, in our hands, no physiologically relevant changes in serum cortisol and DHEA levels after the exercise program could be observed, in line with other studies [48]. However, it is possible that a change in serum cortisol and DHEA levels is only elicited by a more intensive training program, as demonstrated by others for cortisol [39]. Finally, future studies should test different exercise intensities, to define a minimal exercise intensity where effects on the immune and/or neuroendocrine system, for example, cortisol, DHEA, and catecholamines, can be detected.

In conclusion, we confirmed that long-term physical exercise is safe in MS patients [49], as evidenced by no changes observed in the serum levels of proinflammatory mediators following the exercise program. In addition, the annualized relapse rate was not significantly different between the exercise (0.45 per year) and the sedentary group (0.35 per year). However, long-term follow-up of patients participating in physical exercise programs remains necessary. Based on our observations, we conclude a shift towards a tolerogenic profile after physical exercise, suggesting a favourable immunologic effect in MS. Indeed, we demonstrated increased numbers of activated pDC, associated with Tr1 development, and decreased secretion of TLR-induced inflammatory mediators in MS patients following the 12-week combined endurance and resistance exercise program. Noteworthy, whether these immune-modulatory effects occur in every subject undergoing a training program or are specific for MS patients needs to be addressed in future studies. Overall, our results suggest that long-term physical exercise favourably impacts the underlying immunopathogenesis of MS.

## Supplementary Material

Supplementary Appendix: Description of the statistical approach of 12 weeks of combined endurance and resistance training reduces innate markers of inflammation in a randomized controlled clinical trial in patients with multiple sclerosis by Deckx et al.
Supplementary Table 1: Detailed patient characteristics and medication use.

## Figures and Tables

**Table 1:** Clinical details of the study population.

	EX	SED	*P* values
Gender (M/F)	13/16	6/10	0.634
Age ± SEM	47 ± 2	50 ± 3	0.516
BMI ± SEM	24 ± 1	24 ± 1	0.821
EDSS ± SEM	3 ± 0.2	3 ± 0.4	0.155
Type MS (CP/RR)	10/19	5/11	0.826
Medication (untreated/1st-line treatment/2nd-line treatment)	4/16/9	2/8/6	0.908

Results are shown as mean ± SEM. Patients were defined as untreated when a wash-out period of at least 3 months was respected before recruitment in the study. 1st-line treatment: IFN-*β* (Avonex, Betaferon, and Rebif) and glatiramer acetate (Copaxone); 2nd-line treatment: alemtuzumab (Campath), natalizumab (Tysabri), and fingolimod (Gilenya).

EX, exercise group; SED, sedentary control group; M, male; F, female; BMI, body mass index; EDSS, expanded disability status scale; MS, multiple sclerosis; CP, chronically progressive MS; RR, relapsing-remitting MS; SEM, standard error of the mean.

**Table 2:** Serum signaling molecule concentrations are not altered following long-term physical exercise in MS.

Molecule	Group	Time	Mean concentration (pg/mL) ± SEM	*P* values
IL-6	EX	PRE	0.60 ± 0.09	0.434
		POST	0.54 ± 0.09	
	SED	PRE	0.45 ± 0.09	0.341
		POST	0.54 ± 0.09	

IL-10	EX	PRE	0.48 ± 0.09	0.731
		POST	0.50 ± 0.09	
	SED	PRE	0.60 ± 0.19	0.097
		POST	0.31 ± 0.04	

IL-12p70	EX	PRE	0.09 ± 0.01	0.312
		POST	0.12 ± 0.02	
	SED	PRE	0.13 ± 0.03	1.000
		POST	0.13 ± 0.02	

TNF-*α*	EX	PRE	1.07 ± 0.11	1.000
		POST	1.05 ± 0.10	
	SED	PRE	1.37 ± 0.18	0.665
		POST	1.23 ± 0.16	

Caspase-1	EX	PRE	100.48 ± 9.78	0.217
		POST	112.97 ± 9.81	
	SED	PRE	121.61 ± 25.43	1.000
		POST	100.00 ± 14.64	

Molecule	Group	Time	Mean concentration (ng/mL) ± SEM	*P* values

MMP-9	EX	PRE	192.35 ± 23.43	1.000
		POST	174.40 ± 17.84	
	SED	PRE	208.36 ± 25.59	0.368
		POST	176.32 ± 20.86	

DHEA	EX	PRE	16.11 ± 1.65	0.300
		POST	17.06 ± 1.98	
	SED	PRE	16.08 ± 2.17	1.000
		POST	16.18 ± 1.90	

Molecule	Group	Time	Mean concentration (*µ*g/dL) ± SEM	*P* values

Cortisol	EX	PRE	22.17 ± 1.73	**0.047**
		POST	23.73 ± 1.74	
	SED	PRE	20.07 ± 1.00	0.110
		POST	21.49 ± 0.78	

Molecule	Group	Time	Mean molar ratio ± SEM	*P* values

Cortisol/DHEA	EX	PRE	13.78 ± 1.41	0.716
		POST	14.50 ± 1.57	
	SED	PRE	12.18 ± 1.44	0.234
		POST	12.80 ± 1.34	

The serum concentration of signaling molecules was measured with ELISA, and the molar ratio of cortisol to DHEA was calculated. Results are shown as mean concentration or molar ratio ± SEM.

IL, interleukin; TNF, tumour necrosis factor; MMP, matrix metalloproteinase; DHEA, dehydroepiandrosterone; EX, exercise group; SED, sedentary control group; PRE, baseline measurement; POST, measurement after 12 weeks; SEM, standard error of the mean.

**Table 3:** A 12-week exercise program induces a decreased fold change of CCR5^+^ cDC upon LPS and IFN-*γ* stimulation and an increased fold change of HLA-DR^+^ pDC upon IQ stimulation.

Marker	Group	Time	cDC		pDC	
			Mean fold change ± SEM	*P* values	Mean fold change ± SEM	*P* values
CCR5	EX	PRE	0.59 ± 0.06	**0.002**	1.45 ± 0.28	1.000
		POST	0.38 ± 0.06		1.98 ± 0.61	
	SED	PRE	0.51 ± 0.06	1.000	1.67 ± 0.37	0.184
		POST	0.56 ± 0.09		2.08 ± 0.98	

CD86	EX	PRE	2.68 ± 0.45	0.100	12.34 ± 2.55	0.383
		POST	2.12 ± 0.23		9.14 ± 2.11	
	SED	PRE	2.44 ± 0.48	1.000	5.70 ± 1.56	0.767
		POST	2.84 ± 0.69		5.95 ± 2.73	

HLA-DR	EX	PRE	1.01 ± 0.01	0.189	0.90 ± 0.03	**0.008**
		POST	1.01 ± 0.01		0.97 ± 0.02	
	SED	PRE	1.06 ± 0.06	0.439	0.92 ± 0.06	0.609
		POST	1.06 ± 0.03		0.82 ± 0.08	

Blood samples were stimulated overnight with LPS in combination with IFN-*γ*, or IQ. The percentage of CCR5, CD86, and HLA-DR positive cDC and pDC after TLR stimulation was measured using flow cytometry. Mean fold changes are calculated as the ratio of the proportion of CCR5, CD86, and HLA-DR positive cDC and pDC in the stimulated condition to the nonstimulated condition. Results are shown as mean fold change ± SEM.

cDC, conventional dendritic cells; pDC, plasmacytoid dendritic cells; LPS, lipopolysaccharide; IQ, imiquimod; EX, exercise group; SED, sedentary group; PRE, baseline measurement; POST, measurement after 12 weeks; SEM, standard error of the mean.

**Table 4:** Exercise results in decreased secretion of the inflammatory mediators, TNF-*α* and MMP-9, upon LPS and IFN-*γ* stimulation.

Inflammatory mediator	Group	Time	LPS + IFN-*γ*		IQ	
			Mean fold change ± SEM	*P* values	Mean fold change ± SEM	*P* values
IL-1*β*	EX	PRE	89.3 ± 11.5	0.461	2.2 ± 0.2	0.790
		POST	77.1 ± 11.5		2.0 ± 0.2	
	SED	PRE	94.1 ± 19.5	0.258	2.7 ± 0.6	**0.029**
		POST	72.0 ± 16.9		1.8 ± 0.4	

IL-6	EX	PRE	111.0 ± 7.7	1.000	15.7 ± 2.1	1.000
		POST	110.8 ± 6.5		15.9 ± 2.5	
	SED	PRE	103.5 ± 12.8	0.782	16.8 ± 3.1	1.000
		POST	109.7 ± 11.0		14.5 ± 3.4	

IL-12p70	EX	PRE	26.0 ± 7.6	0.313	1.2 ± 0.1	1.000
		POST	15.2 ± 4.1		1.2 ± 0.2	
	SED	PRE	18.1 ± 6.4	1.000	1.6 ± 0.4	0.410
		POST	19.5 ± 6.8		2.2 ± 0.6	

TNF-*α*	EX	PRE	55.1 ± 5.7	**0.028**	1.6 ± 0.1	0.926
		POST	39.2 ± 4.4		1.5 ± 0.1	
	SED	PRE	43.9 ± 8.0	1.000	1.6 ± 0.2	0.168
		POST	46.5 ± 9.3		1.3 ± 0.2	

IFN-*α*	EX	PRE	2.1 ± 0.6	1.000	7.4 ± 1.2	1.000
		POST	2.7 ± 0.9		8.0 ± 1.6	
	SED	PRE	1.9 ± 0.8	1.000	6.7 ± 1.8	0.231
		POST	1.7 ± 0.6		4.4 ± 1.0	

Caspase-1	EX	PRE	8.8 ± 0.7	0.604	4.8 ± 0.7	0.260
		POST	8.0 ± 0.7		3.6 ± 0.4	
	SED	PRE	8.1 ± 1.0	1.000	3.6 ± 0.6	1.000
		POST	6.8 ± 0.8		3.8 ± 0.9	

MMP-9	EX	PRE	7.9 ± 0.8	**0.040**	2.2 ± 0.2	1.000
		POST	6.2 ± 0.5		2.1 ± 0.1	
	SED	PRE	6.5 ± 1.0	0.233	2.0 ± 0.2	1.000
		POST	6.4 ± 0.6		2.0 ± 0.3	

Blood samples were stimulated overnight with LPS in combination with IFN-*γ*, or IQ. Secretion of inflammatory mediators was quantified using ELISA. Mean fold changes are calculated as the ratio of the secreted concentration in the stimulated condition to the nonstimulated condition. Results are shown as mean fold change ± SEM.

LPS, lipopolysaccharide; IQ, imiquimod; IL, interleukin; TNF, tumor necrosis factor; IFN, interferon; MMP, matrix metalloproteinase; EX, exercise group; SED, sedentary control group; PRE, baseline measurement; POST, measurement after 12 weeks; SEM, standard error of the mean.

**Table 5:** Circulating Treg subsets are not affected by long-term physical exercise in MS.

Treg subset	Group	Time	Mean % Treg in CD4^+^ T cell population ± SEM	*P* values
CD25^hi^FoxP3^+^	EX	PRE	0.62 ± 0.11	0.083
		POST	1.12 ± 0.41	
	SED	PRE	1.00 ± 0.30	0.782
		POST	1.69 ± 0.85	

IL-10^+^ (Tr1)	EX	PRE	0.22 ± 0.05	1.000
		POST	0.20 ± 0.02	
	SED	PRE	0.33 ± 0.09	0.796
		POST	0.50 ± 0.26	

TGF-*β* ^+^ (Th3)	EX	PRE	0.15 ± 0.02	1.000
		POST	0.19 ± 0.09	
	SED	PRE	0.17 ± 0.04	0.056
		POST	0.12 ± 0.08	

The percentage of circulating Treg subsets was determined using flow cytometry. Results are shown as mean percentage ± SEM.

Treg, regulatory T cell; FoxP3, forkhead box P3; IL, interleukin; Tr1, type 1 regulatory T cell; TGF, transforming growth factor; Th3, T helper 3 cell; EX, exercise group; SED, sedentary control group; PRE, baseline measurement; POST, measurement after 12 weeks; SEM, standard error of the mean.
