# Supplementary material for: 12 Weeks of Combined Endurance and Resistance Training Reduces Innate Markers of Inflammation in a Randomized Controlled Clinical Trial in Patients with Multiple Sclerosis
Source: Mediators Inflamm. 2016 Jan 20;2016:6789276. doi: 10.1155/2016/6789276 (PMC4745915; doi:10.1155/2016/6789276)
Supplement: Supplementary file 1 — Supplementary Appendix: Description of the statistical approach of 12 weeks of combined endurance and resistance training reduces innate markers of inflammation in a randomized controlled clinical trial in patients with multiple sclerosis by Deckx et al. Supplementary Table 1: Detailed patient characteristics and medication use. [file 6789276.f1.doc]

**Supplementary Materials:**

**Appendix to 12 weeks of combined endurance and resistance training reduces innate markers of inflammation in a randomized controlled clinical trial in patients with multiple sclerosis by Deckx et al. Statistical approach**

With this study we aimed to investigate the effect of a 12-week combined endurance and resistance exercise program on cellular and molecular mediators of innate immunity in patients with multiple sclerosis (MS). For this, MS patients were randomized by sealed envelope following a 3:2 ratio to an exercise intervention group (n = 38) or a sedentary control group (n = 25). We hypothesized that an effect of time, i.e. the 12-week exercise intervention, could be observed in the trained group of patients but not in the sedentary group of patients. Therefore, linear mixed models were used to analyze repeated measures data.

Whereas two-repeated measures analysis and 2-by-2 factorial designs can evaluate the expected interaction effect between time and group, i.e. trained and sedentary MS patients, the general mixed model methodology was chosen for its ability to study the impact of multiple covariates in combination with the time effect, i.e. including covariate by time interactions. A model was built stepwise per outcome variable, starting from a univariate model with time as the only fixed effect. Step by step new models were constructed by adding other fixed effect variables including, group, MS type, gender, age, body mass index (BMI), expanded disability status scale (EDSS) and MS-specific medication. A *P* value < 0.10 (F test) was used as the threshold for retaining a fixed effect during model building to decrease the chance of missing a significant effect in the final model. Subsequently, possible interaction effects between the retained fixed effects were assessed, and also retained if *P* < 0.10. Thus, linear mixed models can evaluate the expected interaction between time and group, possible interaction effects between time and other covariates as well as baseline influences of these other covariates. In the manuscript, we only reported main and interaction effects of time, since our main interest was the effect of time, i.e. the 12-week exercise intervention. Nevertheless, baseline influences of covariates are interesting and could lead to alternative treatment modalities. For this, we used mixed models rather than pairwise regression analysis. Indeed, pairwise regression analysis would be an alternative approach but cannot be used to study baseline influences of covariates. Note that their equivalence for studying the effect of time can be easily proven mathematically.

Furthermore, with significant interaction effects of time, post-hoc analysis were performed using the Bonferroni correction to correct for multiple testing. Nonetheless, post-hoc analysis for both the exercise and the sedentary group was performed even if the group by time interaction was not significant. First of all, we were a priori interested in this subgroup analysis. We hypothesized that an effect of time, i.e. the 12-week exercise intervention, could be observed in the trained group of patients but not in the sedentary group of patients. This hypothesis was supported by previous findings reported by others indicating that long-term physical exercise is able to modulate endocrine and immune parameters in healthy populations [Walsh (2011) Position statement. Part one: Immune function and exercise. Exercise Immunology Review]. Second, we used the conservative Bonferroni correction to account for multiple testing, for the evaluation of time-mediated effects, thereby reducing the chance of detecting false positive results. Overall, this justifies our decision to proceed with subgroup analysis after a non-significant interaction value. Noteworthy, subgroup analysis was only performed in trained and sedentary MS patients, and not for MS type, gender and MS-specific medication, unless a significant interaction effect with time was found.

In addition, several patients did not complete the study. Some patients discontinued the intervention because they had mental or health problems, quit the study by own chose, or experienced a relapse during the study. Other patients were lost to follow-up because they were not able to donate blood or did not show up at the end of the study. At the end of the study, 29 and 16 patients from the exercise group and the sedentary group were included in the analysis, respectively. To determine safety of the intervention, patients who relapsed during the study were noted and the annualized relapse rate was calculated for both groups. The annualized relapse rate was not significantly different between the exercise (0.45 per year) and the sedentary group (0.35 per year). In addition, the ratio of patients who discontinued the intervention and the ratio of patients who were lost to follow-up were not significantly different between the exercise and the sedentary group (Table 1).

**Table 1. Drop-out ratio for the exercise and the sedentary group**

|  | Exercise group | Sedentary group | *P* values |
| --- | --- | --- | --- |
| Relapse | 4 out of 38 patients | 2 out of 25 patients | 0.743 |
| Discontinuation of intervention | 5 out of 38 patients | 6 out of 25 patients | 0.275 |
| Loss to follow-up | 4 out of 38 patients | 3 out of 25 patients | 0.858 |

Patients discontinued the intervention because they had mental or health problems, quit the study by own chose, or experienced a relapse during the study. Patients were lost to follow-up because they were not able to donate blood or did not show up at the end of the study.

Furthermore, since our main interest is the effect of time, i.e. the 12-week exercise intervention, we did not include baseline measurements of patients who did not complete the study. Because the intervention was not found to be responsible for the drop-out reason, we can assume data are missing completely at random and thus that the exclusion of missing values does not lead to bias. In support of this, no differences were found when comparing the baseline characteristics of patients in both groups to the characteristics after excluding data (Table 2).

**Table 2. Comparison of the baseline characteristics to the characteristics after exclusion**

|  | Exercise Group | | | Sedentary Group | | |
| --- | --- | --- | --- | --- | --- | --- |
|  | All patients (n=38) | Analyzed patients (n=29) | *P* values | All patients (n= 25) | Analyzed patients (n=16) | *P* values |
| Gender (M/F) | 15/23 | 13/16 | 0.666 | 9/16 | 6/10 | 0.925 |
| Age ± SEM | 47 ± 2 | 47 ± 2 | 0.783 | 49 ± 2 | 50 ±3 | 0.711 |
| BMI ± SEM | 25 ± 1 | 24 ± 1 | 0.347 | 26 ± 1 | 24 ± 1 | 0.159 |
| EDSS ± SEM | 3 ± 0.2 | 3 ± 0.2 | 0.924 | 3 ± 0.3 | 3 ± 0.4 | 0.606 |
| Type MS (CP/RR)* | 14/23 | 10/19 | 0.783 | 5/18 | 5/11 | 0.516 |
| Medication (untreated/1^st^-line treatment/2^nd^-line treatment)* | 6/21/11 | 4/16/9 | 0.803 | 2/16/6 | 2/8/6 | 0.677 |

Patients were defined as untreated when a wash-out period of at least 3 months was respected before recruitment in the study. 1^st^-line treatment: IFN-β treatment (Avonex®, Betaferon®, Rebif®) and glatiramer acetate (Copaxone®); 2^nd^-line treatment: alemtuzumab (Campath®), natalizumab (Tysabri®) and fingolimod (Gilenya®). Results are shown as mean ± SEM. *data not available for every patient.

Abbreviations used: M, male; F, female; BMI, body mass index; EDSS, expanded disability status scale; MS, multiple sclerosis; CP, chronic-progressive MS; RR, relapsing-remitting MS; SEM, standard error of the mean.

Moreover, for three outcome variables that were significant in our analysis we conducted a sensitivity analysis by including the baseline measurements of patients who did not complete the study, i.e. drop-outs, in the mixed model. Note that doing so the missingness mechanism is relaxed to missing at random, i.e. drop-out is allowed to depend on observed measurements but not on unobserved measurements. The advantage of the latter model is a reduction in loss of efficiency since the analysis is based on all available cases. As shown in Table 3, this did not lead to different results and conclusions, in support of our initial analysis. The results in Table 3 do not show an apparent loss of efficiency when excluding the baseline measurements as is expected for the interaction effects. Indeed, baseline measurements alone do not provide any information about time trends. For more details on the validity of our analysis we refer to [Molenberghs and Kenward (2007) Missing data in clinical studies. Wiley].

**Table 3. Analysis of interaction between time and group without and with inclusion of drop-outs**

|  | Without drop-outs | | With drop-outs | |
| --- | --- | --- | --- | --- |
|  | Interaction | Post-hoc | Interaction | Post-hoc |
| Number of pDC | *P*=0.017 | EX: *P*=0.010  SED: *P*=0.759 | *P*=0.008 | EX: 0.007  SED: *P*=0.652 |
| Number of CD80^+^ pDC | *P*=0.015 | EX: *P*<0.001  SED: *P*=0.370 | *P*=0.016 | EX: *P*<0.001  SED: *P*=0.856 |
| Fold change of % HLA-DR^+^ pDC after IQ stimulation | *P*=0.023 | EX: *P*=0.008  SED: *P*=0.609 | *P*=0.021 | EX: *P*=0.008  SED: *P*=0.498 |

Interaction effects between time and group are shown. Post-hoc analyses in both groups were performed using the Bonferroni correction to correct for multiple testing.

Moreover, the significance level of baseline influences of other covariates than time and group did not change with inclusion of the drop-outs for 3 outcome variables that were significant (Table 4).

**Table 4. Analysis of baseline influences of covariates** without and with inclusion of drop-outs

|  | Without drop-outs | | With drop-outs | |
| --- | --- | --- | --- | --- |
|  | Covariates | *P* values | Covariates | *P* values |
| Number of pDC | Type MS  Gender | 0.036  0.081 | Type MS  Gender | 0.012  0.115 |
| Number of CD80^+^ pDC | No covariates included in the model | / | No covariates included in the model | / |
| Fold change of % HLA-DR^+^ pDC after IQ stimulation | No covariates included in the model | / | No covariates included in the model | / |

Covariates that were included during model building are shown.

**Supplementary Table 1. Study subjects and disease characteristics**

| **Exercise group (n=29)** | | | | | | | **Sedentary group (n=16)** | | | | | | |
| --- | --- | --- | --- | --- | --- | --- | --- | --- | --- | --- | --- | --- | --- |
| **ID** | **Type MS** | **EDSS** | **MS medication** | **Age** | **Gender** | **BMI** | **ID** | **Type MS** | **EDSS** | **MS medication** | **Age** | **Gender** | **BMI** |
| **UPN202** | CP | 6 | None | 49 | F | 19 | **UPN212** | RR | 6 | Copaxone® | 60 | M | 28 |
| **UPN205** | RR | 2 | Avonex® | 50 | M | 25 | **UPN216** | RR | 2 | Avonex® | 52 | F | 20 |
| **UPN207** | CP | 3 | None | 58 | F | 25 | **UPN219** | CP | 2.5 | Avonex® | 65 | M | 24 |
| **UPN208** | CP | 4.5 | None | 49 | M | 22 | **UPN225** | RR | 2.5 | Rebif® | 52 | F | 17 |
| **UPN209** | CP | 2.5 | Betaferon® | 52 | F | 24 | **UPN230** | CP | 6 | Rebif®, Campath® | 55 | F | 25 |
| **UPN210** | CP | 3.5 | Rebif® | 59 | M | 24 | **UPN237** | CP | 4 | Campath® | 35 | M | 22 |
| **UPN211** | RR | 1.5 | Tysabri® | 38 | F | 24 | **UPN239** | CP | 5.5 | Rebif® | 60 | M | 23 |
| **UPN223** | RR | 2.5 | Rebif® | 54 | M | 21 | **UPN240** | RR | 3 | Rebif®, Campath® | 41 | F | 20 |
| **UPN224** | RR | 1.5 | Tysabri® | 27 | M | 25 | **UPN304** | RR | 4 | None | 36 | M | 21 |
| **UPN227** | RR | 3 | Avonex® | 32 | M | 23 | **UPN308** | CP | 4 | None | 54 | M | 27 |
| **UPN231** | RR | 2.5 | Tysabri® | 45 | F | 22 | **UPN311** | RR | 2 | Avonex® | 56 | F | 25 |
| **UPN232** | CP | 4 | Tysabri®, Avonex® | 45 | F | 19 | **UPN313** | RR | 1 | Avonex® | 42 | F | 24 |
| **UPN234** | RR | 4 | Rebif®, Betaferon® | 29 | M | 25 | **UPN317** | CP | 3 | Gilenya® | 62 | F | 25 |
| **UPN235** | RR | 2.5 | Rebif®, Copaxone® | 59 | F | 27 | **UPN326** | RR | 1.5 | Tysabri® | 40 | F | 25 |
| **UPN236** | RR | 2.5 | Avonex® | 41 | F | 21 | **UPN332** | RR | 2.5 | Avonex® | 54 | F | 27 |
| **UPN238** | CP | 4 | Copaxone® | 51 | F | 32 | **UPN334** | RR | 1.5 | Tysabri® | 33 | F | 28 |
| **UPN240** | RR | 3 | Rebif®, Campath® | 41 | F | 20 |  |  |  |  |  |  |  |
| **UPN241** | RR | 3 | Betaferon® | 56 | M | 28 |  |  |  |  |  |  |  |
| **UPN242** | RR | 5.5 | Betaferon®, Tysabri® | 58 | F | 26 |  |  |  |  |  |  |  |
| **UPN305** | RR | 2 | Tysabri® | 44 | F | 22 |  |  |  |  |  |  |  |
| **UPN309** | CP | 4 | Avonex® | 50 | M | 22 |  |  |  |  |  |  |  |
| **UPN314** | RR | 2 | Avonex® | 54 | F | 20 |  |  |  |  |  |  |  |
| **UPN321** | CP | 2.5 | Copaxone® | 54 | F | 23 |  |  |  |  |  |  |  |
| **UPN324** | CP | 5 | Rebif® | 54 | M | 23 |  |  |  |  |  |  |  |
| **UPN328** | RR | 3 | Gilenya® | 34 | M | 23 |  |  |  |  |  |  |  |
| **UPN330** | RR | 1.5 | Tysabri® | 29 | M | 24 |  |  |  |  |  |  |  |
| **UPN333** | RR | 4 | Gilenya® | 52 | F | 22 |  |  |  |  |  |  |  |
| **UPN335** | RR | 1.5 | Rebif® | 58 | M | 24 |  |  |  |  |  |  |  |
| **UPN336** | RR | 2.5 | None | 49 | F | 31 |  |  |  |  |  |  |  |
|  | CP/RR:  10/19 | Mean: 3  SEM: 0.2 |  | Mean: 47  SEM: 2 | M/F:  13/16 | Mean: 24  SEM: 1 |  | CP/RR:  5/11 | Mean: 3  SEM: 0.4 |  | Mean: 50  SEM: 3 | M/F:  6/10 | Mean: 24  SEM: 1 |

Abbreviations used: MS, multiple sclerosis; RR, relapsing-remitting MS; CP, chronically-progressive MS; UPN, unique patient number; EDSS, expanded disability status scale; EX, exercise group; SED, sedentary group. 1^st^ line treatment: IFN-β (Avonex®, Betaferon®, Rebif®) and glatiramer acetate (Copaxone®). 2^nd^ line treatment: alemtuzumab (Campath®), natalizumab (Tysabri®) and fingolimod (Gilenya®). Patients were defined as untreated when a wash-out period of at least 3 months was respected before recruitment in the study.
